# Supplementary material for: Predicting visual function by interpreting a neuronal wiring diagram
Source: Nature. 2024 Oct 2;634(8032):113–23. doi: 10.1038/s41586-024-07953-5 (PMC11446822; doi:10.1038/s41586-024-07953-5)
Supplement: Supplementary file 6 — CRF and ERF predictions for individual TmY4 and TmY9 cells. Analogous to Supplementary Data 3, but for TmY target types. Shown are the top four monosynaptic pathways, the strongest pathway passing through each of the top ten intermediary types (ranking from Extended Data Fig. 7), and the trisynaptic pathway Tm1–TmY–Dm3–TmY (see the section entitled Prediction of spatial normalization). [file 41586_2024_7953_MOESM6_ESM.zip › DataS4/TmY9q/720575940616665942.pdf]

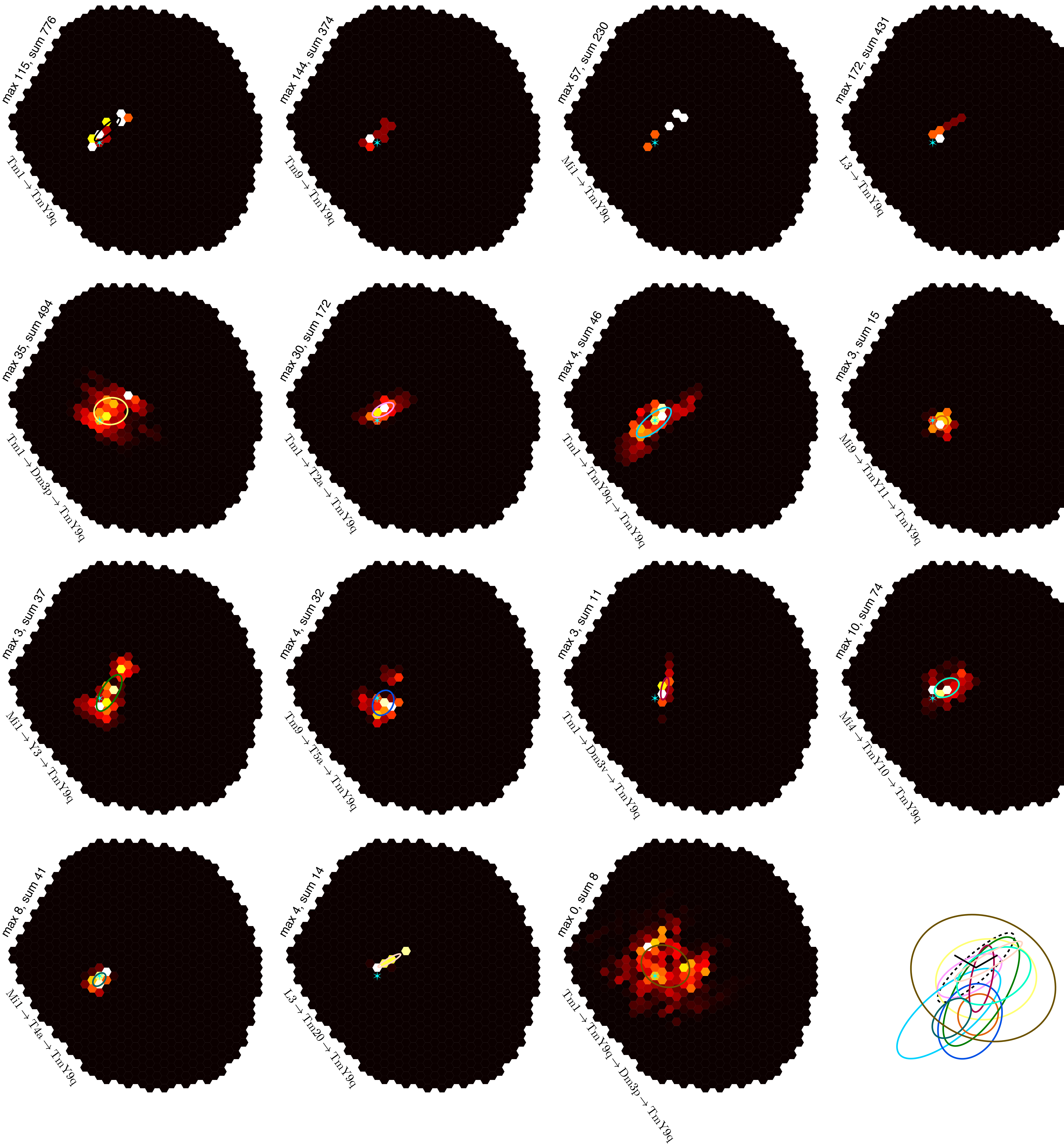

max 115, sum 776
Tm1 → TmY9q
max 144, sum 374
Tm9 → TmY9q
max 57, sum 230
Mi1 → TmY9q
max 172, sum 431
L3 → TmY9q
max 35, sum 494
Tm1 → Dm3p → TmY9q
max 30, sum 172
Tm1 → T2a → TmY9q
max 4, sum 46
Tm1 → TmY9q → TmY9q
max 3, sum 15
Mi9 → TmY11 → TmY9q
max 3, sum 37
Mi1 → Y3 → TmY9q
max 4, sum 32
Tm9 → T5a → TmY9q
max 3, sum 11
Tm1 → Dm3v → TmY9q
max 10, sum 74
Mi4 → TmY10 → TmY9q
max 8, sum 41
Mi1 → T4a → TmY9q
max 4, sum 14
L3 → Tm20 → TmY9q
max 0, sum 8
Tm1 → TmY9q → Dm3p → TmY9q
